# Broccoli plants exposed to the combined threat of climate change and bacterial infection

**DOI:** 10.1186/s12870-026-08704-6

**Published:** 2026-04-09

**Authors:** María Luisa Pérez-Bueno, Matilde Barón, Mónica Pineda

**Affiliations:** 1 Stress, Development and Signalling in Plants, Estación Experimental del Zaidín, Spanish National Research Council, Granada, Spain; 2 Department of Plant Physiology, Facultad de Ciencias, University of Granada, Granada, Spain

**Keywords:** Black rot, Fluorescence imaging, Representative concentration pathway (RCP), Thermography, *Xanthomonas campestris* pv. *campestris*

## Abstract

**Background:**

Climate change is expected to intensify plant diseases by altering both host physiology and pathogen behaviour, posing a growing risk to global food security. *Brassica* crops such as broccoli (*Brassica oleracea* var. *italica*), a key vegetable for human nutrition, are particularly vulnerable to black rot caused by *Xanthomonas campestris* pv. *campestris* (Xcc). To assess how future climates may influence this particular pathosystem, broccoli plants were cultivated under three environmental regimes: current climate conditions (CCC) and two late-century projections—an intermediate scenario (RCP 4.5: +3 °C and 650 ppm CO_2_) and an extreme scenario (RCP 8.5: +6 °C and 1000 ppm CO_2_). Plants were inoculated with Xcc races 1 and 4.

**Results:**

The moderate scenario (RCP 4.5) did not negatively affect plant fitness, whereas severe future conditions (RCP 8.5) markedly reduced vegetative growth and photosystem II efficiency. Black rot severity and bacterial growth kinetics depended on both pathogen race and environmental conditions: race 4 was most aggressive under CCC and RCP 4.5, while race 1 gained virulence under RCP 8.5 at later infection stages. Regardless of race, infection increased leaf temperature and lipid peroxidation and stimulated the accumulation of phenolics and other antioxidant metabolites, indicating a shift toward defence-related secondary metabolism. These metabolic changes mitigated oxidative damage but did not prevent the decline in photosynthetic performance under climate conditions.

**Conclusions:**

These findings demonstrate that future climate scenarios will not only compromise broccoli physiology but also alter the performance of Xcc races selectively.

**Supplementary Information:**

The online version contains supplementary material available at 10.1186/s12870-026-08704-6.

## Introduction

The global climate is experiencing a series of unprecedented changes, characterized by rising temperatures, modified rainfall trends, and more frequent intense meteorological events. These alterations impose stress conditions on crops, directly affecting plant physiology and development by disrupting fundamental processes such as photosynthesis, water uptake, germination, growth, and flowering [1]. Thus, global warming has profound implications for agriculture, affecting crop yield [2, 3]. Climate change is expected to alter the traditional growing seasons as well as the optimal geographic regions for certain crops, causing the need for new varieties that could be better adapted to the changing climate or even adopting different crops better suited to new conditions. Consequently, climate change could drastically affect crop production and yields, compromising global food availability and security [4]. Among crop species, *brassicas* are important for both human diets and livestock feed because of the high nutritional value of their products (leaves, flowers, oilseeds). For this reason, multiple researches have addressed the impact of climate change on broccoli (*Brassica oleracea* var. *italica*) plants [5–8].

Environmental conditions not only impact on crops, but also on the microorganisms in the rhizosfere and phyllosphere, affecting their benefitial and pathogenic relations with plants. Thus, warmer temperatures and changing climatic conditions may create more favorable environments for certain diseases, facilitating the accumulation, migration and spread of pests and pathogens to new regions, increasing pressure on previously unaffected crops [9]. Increasing outbreaks of plant diseases will further threaten food security worldwide. Consequently, it is essential to deepen our knowledge of the effects of future climate on crop physiology and their interactions with pathogens and pests [10]. However, the body of available research examining the effects of climate change on plant pathology is limited, particularly in *brassicas*.

Xcc is responsible for causing black rot, one of the most destructive diseases impacting *brassica* crops globally [11, 12]. Black rot is characterised by V-shaped, necrotic lesions on the leaf margins that radiate towards the leaf edges. Xcc can colonise the apoplast as well as vascular tissues, causing systemic wilting and chlorosis of leaves [13]. As the disease progresses, plants may exhibit stunted growth and premature senescence and eventually die [14, 15]. A recent increase in the incidence of plant diseases caused by species of the genus *Xanthomonas* has been observed, which has been linked, among other factors, to climate change [16]. Moreover, the surveillance of Xcc in the phyllosphere and rhizosphere of several crops that may act as bacterial reservoirs is notably influenced by climatic variables [17]. Pineda et al. [18] analysed the impact of environmental conditions on *Xanthomonas campestris* pv. *campestris* (Xcc)-infected broccoli plants grown under current climate conditions (CCC) compared to those grown under climate change conditions: RCP 4.5 and RCP 8.5 [representative concentration pathways established by the Intergovernmental Panel on Climate Change (IPCC) for years 2081–2100; [19]; i.e., projections of future climate, representing an intermediate and an extreme scenario of global warming, respectively. The evolution and severity of symptoms was quantified using imaging techniques based on reflectance and leaf temperature. Under current climate conditions (CCC) and under the intermediate RCP 4.5, race 4 was more virulent on broccoli plants than race 1. In contrast, race 1 was the most virulent race at longer infection times under extreme ambient conditions or RCP 8.5. Similar results were found for oilseed rape plants infected with Xcc [20].

A comprehensive understanding of how global warming influences plant pathogenesis is essential for designing targeted strategies to mitigate its detrimental effects on broccoli production and strengthen crop resilience. This study aimed to explore the intricate interplay between broccoli plant physiology and the pathogenic bacterium Xcc, and to determine how climate change could affect the development of black rot in *brassicas*. The mechanisms underlying broccoli responses to both abiotic stressors associated with climate change and the biotic stress induced by bacterial infection were studied using a physiological approach. Thus, the effects of Xcc infection in broccoli plants grown under climate change projections RCP 4.5 and RCP 8.5 were assessed in terms of photosynthetic activity and transpiration, antioxidant metabolism, and secondary metabolism. These analyses were complemented by the in vivo kinetics of bacterial growth in leaf tissues under the three climatic conditions.

## Materials and methods

### Plant growth under current and climate change conditions

The experimental design for broccoli *Brassica oleracea* var. *italica* ‘Calabrese Natalino’ (Semillas Fitó, Barcelona, Spain) cultivation was based on climate change projections from the IPCC 5th Assessment Report (AR5), with data downscaled by AEMet (State Meteorological Agency) for the Región de Murcia, the largest broccoli-producing area in Spain. Two climate change scenarios were considered: an intermediate projection (RCP 4.5) and an extreme projection (RCP 8.5) for the period 2081–2100 [19]. For each trial, plants were sown and grown under CCC, RCP 4.5, or RCP 8.5 conditions, as follows: five or six broccoli seeds were placed in 125 cm³ seed trays (5 × 5 × 5; height × width × depth, cm) filled with soil (Compo Sana Universal, Münster, Germany). The substrate contained peat (decomposition level H3–H8), perlite, lime, NPK fertiliser, silicon phosphate and micronutrients. After 7 days, seedlings at BBCH stage 09 (cotyledons fully emerged, first true leaf not yet visible; [21]) were transplanted into approximately 1 L pots (9 cm base diameter, 13 cm top diameter and 10.5 cm height) containing a 2:1 (v/v) mixture of soil and coconut fibre (Projar, Valencia, Spain), with one plant per pot. No additional fertilisation was applied during the experimental period. Plants were irrigated daily and maintained under non-limiting water conditions. The conditions for each climatic treatment are detailed in Table 1.

To contextualize the atmospheric evaporative demand across climate scenarios, the vapour pressure deficit (VPD) was calculated using the air temperature (T) and relative humidity values reported in Table 1, according to the following equations:

1 $$\mathrm{VPD}\;=\mathrm{e}_\mathrm{s}(T)\;(1-\mathrm{relative}\;\mathrm{humidity}/100)$$

2 $$\mathrm{e}_{\mathrm{s}}(\mathrm{T})\;=\;0.6108\mathrm{exp}[(17.27 \cdot \mathrm{T})/(\mathrm{T}+237.3)$$

**Table 1:** Climatic conditions used for broccoli cultivation according to IPCC AR5 and regionalised for Región de Murcia for years 2081–2100. CCC, current climate conditions; RCP 4.5 and RCP 8.5, Representative Concentration Pathways 4.5 and 8.5, respectively. The day and night temperatures correspond to the averaged values during the growing season. Vapour Pressure Deficit (VPD) was calculated according to Eqs. (1) and (2)

	Climatic treatment		
	CCC	RCP 4.5	RCP 8.5
Day temperature (ºC)	31	34	37
Night temperature (ºC)	17	20	23
CO_2_ (ppm)	408	650	1000
Photoperiod (h day/night)	18/6	18/6	18/6
Light intensity (mol photon m^− 2^ s^− 1^ of PAR light)	200	200	200
Relative humidity (%)	60	60	60
Day/night Vapour Pressure Deficit (VPD) (kPa)	1.80/0.78	2.12/0.94	2.51/1.12

### Bacterial growth and inoculation

*Xanthomonas campestris* pv *campestris* (Xcc) races used in this study included race 1 (HRI 3811; originally isolated from *B. oleracea* in the United States) and race 4 (HRI 1279 A; originally isolated from *B. oleracea* var. *capitata* in the United Kingdom). Both races were stored at -80 °C in a 50% glycerol solution until the moment of use. Original isolates are preserved in the Warwick University (Coventry, United Kingdom) [11]. The day before plant inoculation, Xcc races 1 and 4 were grown in LB (Luria-Bertani growth media: 10 g/L tryptone; 5 g/L yeast extract; 5 g/L sodium chloride; 14 g/L bacteriological agar; pH 7.0) plates for 24 h at 28 °C. Bacterial suspensions were constituted in sterile MgCl_2_ 10mM at 10^8^ colony forming units per millilitre (cfu mL^− 1^) by setting the optical density of the bacterial suspension at 600 nm to 0.1 [18].

Plant were inoculated at BBCH stage 14 (four true leaves, leaf pairs or whorls unfolded; [21]). Thus, plants aged four weeks (in the case of CCC or RCP 4.5) and five weeks (under RCP 8.5) were inoculated with the pathogen on the third leaf by mechanically injuring four secondary veins using rat tooth tweezers that had been pre-soaked in the respective bacterial suspension. Additionally, controls were mock-inoculated with sterile 10mM MgCl_2_. Each assay included twelve plants per bacterial race or mock-controls in the case of experiments conducted under CCC and RCP 4.5. Conversely, a total of four plants per treatment were used in the experiments performed under the RCP 8.5 scenario. At least two independent trials were conducted for each climatic scenario, yielding consistent results. 

### In vivo bacterial growth kinetics

Bacterial density was quantified by recovering bacteria from six leaf discs (4.15 cm^2^ each) grounded in 10 mM MgCl_2_. Serial dilutions were plated on LB agar, and cfus per leaf area were counted after 48 h, according to [22]. Samples were collected at 0, 1, 2, 3, 6 and 9 dpi (days post-inoculation).

### Quantification of leaf redox state, pigments and soluble phenolics

Samples were collected using 4.15 cm^2^ leaf discs (1.54 cm^2^ in the case of broccoli plants cultivated at RCP 8.5) practiced around the inoculation point of broccoli plants after 3 and 6 dpi. For each environmental condition, six different leaves from separate plants were sampled and weighted for every treatment and measurement, or five plants in the case of RCP 8.5. Fresh weight (FW) per leaf area was determined based on the measured sample weights. Leaf discs were promptly immersed in liquid nitrogen after collection and preserved at − 80 °C. Spectrophotometric readings were obtained with a Shimadzu UV-1800 instrument (Shimadzu Corporation, Tokyo, Japan). The leaf redox state was assessed by measuring the total antioxidant capacity (TAA) according to [23], as well as the lipid peroxidation according to [24]. The leaf total content on chlorophylls (Chl) was evaluated according to [25]. Total content on soluble phenolics, flavonoids, ortho-diphenols and phenylpropanoid glycosides (PPGs) were also determined according to [26–28] and [29], respectively. Determinations were referred to leaf area.

### Metabolic profile analysis

The content on sucrose and the acids citric, caffeic and ferulic was analysed by gas chromatography followed by mass spectrometry (GS-MS) according to [30] using a Varian (now Bruker Corporation, Billerica, MA, USA) 450GC 240MS system for GC-MS [31]. Six leaf disc samples were analysed from plants grown at CCC and RCP 4.5 (five leaf disc samples from plants grown at RCP 8.5). Each leaf disc was pierced in tissue surrounding the inoculation point. Samples were collected at 3 and 6 dpi, weighted, pulverized in liquid nitrogen and stored at -80 °C. Determinations were referred to leaf area.

### Imaging techniques

Inoculated attached leaves were analysed by imaging techniques at 3 and 6 dpi. Additionally, thermal images were also taken at 1, and 2 dpi. Twelve inoculated leaves were measured per treatment and dpi (four under RCP 8.5 conditions). In the case of Chl-FI, eight inoculated leaves were measured per treatment and dpi. Average values ± standard errors of every physiological parameter were obtained from the image of regions around the inoculation site of each plant and treatment.

Whole-leaf thermal imaging was conducted using a FLIR A305sc camera (FLIR Systems, Wilsonville, OR, USA) controlled by FLIR ResearchIR v. 3.4 software, according to [22]. For a better comparison between climatic treatments, the parameter T_L_ – T_A_ (leaf temperature, T_L_, corrected by ambient temperature, T_A_) was computed [32].

Leaf autofluorescence excited by UV light was measured using a customized Open FluorCam FC 800-O (Photon Systems Instruments, Brno, Czechia), according to [33]. The camera collects black and white images of the emitted fluorescence at the blue (F440) and green 520 nm (F520) regions of the spectrum. The FluorCam v. 7.1.0.3 software provides a false colour scale to the images.

Chl-FI was used to assess the photosynthetic performance of photosystem II (PSII). Images were acquired using an Open FluorCam 700 MF (Photon System Instruments), controlled by FluorCam v. 5 software. Recordings were carried out according to [34]. Prior to measurements, plants were dark-adapted for 30 minutes. For each inoculated leaf, calculated parameters included the maximum quantum yield of PSII [F_V_/F_M_ = (F_M_-F_0_)/F_M_], the effective PSII quantum yield [Φ_PSII_ = (F_M_’-F_t_)/F_M_’] and the non-photochemical quenching [NPQ = (F_M_-F_M_’)/F_M_’] [35]. Both Φ_PSII_ and NPQ were calculated for the steady-state.

### Data mining and statistical analysis

Graphs were plotted using Microsoft Excel for Microsoft 365 MSO (Microsoft, United States). Statistical analyses were conducted using SPSS version 28.0 software (IBM Corporation, Armonk, NY, USA). Experiments at each environmental condition were performed twice, yielding comparable outcomes. Data displayed in figures are representative results for each environmental condition.

Outliers were removed from each dataset. Bivariate correlations with confidence interval were carried out for the data (*p* ≤ 0.001). Then, normality of the data was verified by the Shapiro–Wilk test, whereas homogeneity of variance was assessed by the Levene test. Multiple comparisons were performed by analysis of variance (ANOVA) followed by Tukey’s HSD using SigmaPlot v. 16 (Graffiti LLC, Palo Alto, CA, USA). Principal component analysis (PCA) and hierarchical clustering of principal components (HCPC) were performed on normalised and standardised variables using RStudio version 2024.12.1 (RStudio, Inc., Boston, MA, USA) based on R 4.3.2. The selected parameters for PCA and subsequent HCPC were determined by ensuring no redundancy and maximising the explained variance.

## Results

### Timing and severity of black rot in broccoli plants depends on the Xcc race and the environmental conditions

**Fig. 1:**
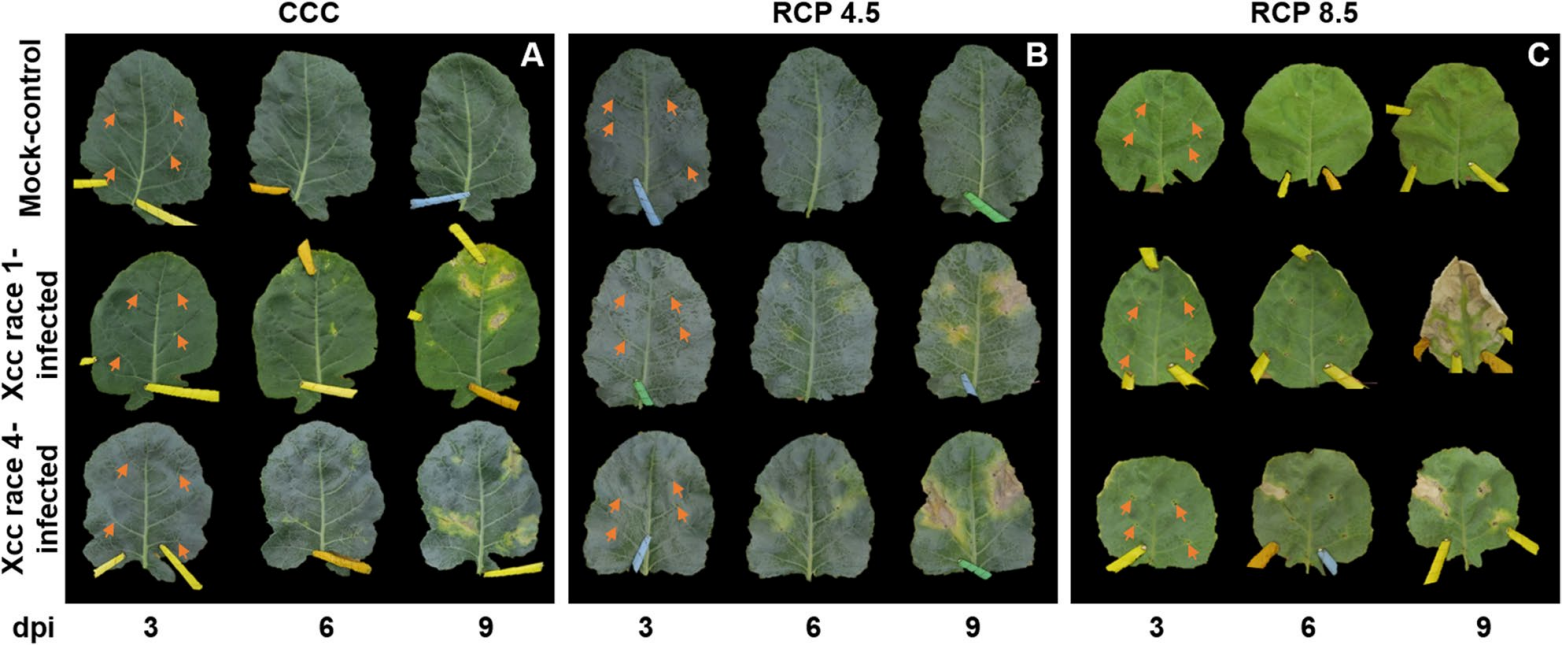
Symptomatology. RGB images of broccoli leaves from plants grown under current climate conditions (CCC; panel **A**) and representative concentration pathways RCP 4.5 and RCP 8.5 (intermediate and extreme climate change conditions, respectively; panels **B** and **C**, respectively), and infected with race 1 or 4 of *Xanthomonas campestris* pv. *campestris* (Xcc). Dpi: days post-inoculation. Arrows indicate inoculation points

Broccoli plants grown under CCC, RCP 4.5 or RCP 8.5 conditions were inoculated with Xcc race 1 or 4 at a concentration of 10^8^ cfu/mL, and the progression of symptoms was monitored from 1 to 9 dpi (Fig. 1). Remarkably, there were no symptoms of infection before 3 dpi under any of the growth conditions tested. Mock-control leaves only exhibited lesions caused by clipping.

Under CCC and RCP 4.5 conditions, Xcc produced chlorosis followed by gradual necrosis around the inoculation site (marked with arrows in Fig. 1). The race that produced the most severe symptoms was Xcc race 4, which induced chlorosis at the clipping site as early as 3 dpi, becoming more visible at 6 dpi. Chlorosis around the inoculated area appeared at 6 dpi, and the V-shaped lesions at 9 dpi. In comparison, Xcc race 1 caused similar symptoms but developed more slowly, with a delay of two days. The RCP 8.5 conditions subjected plants to environmental stress, resulting in an adverse effect on vegetative growth, leading to stunting and symptoms of early senescence. In addition, leaves exhibited reduced dimensions and increased thickness compared with those of plants grown under CCC or RCP 4.5 environments. Under RCP 8.5 conditions, the progress of the infection of both Xcc races resembled that observed for Xcc race 4 under CCC. However, Xcc race 1 under extreme climate conditions appeared more virulent than Xcc race 4 at 9 dpi. 

### The *in planta* growth of Xcc races 1 and 4 is affected differentially by ambient conditions resembling climate change

**Fig. 2:**
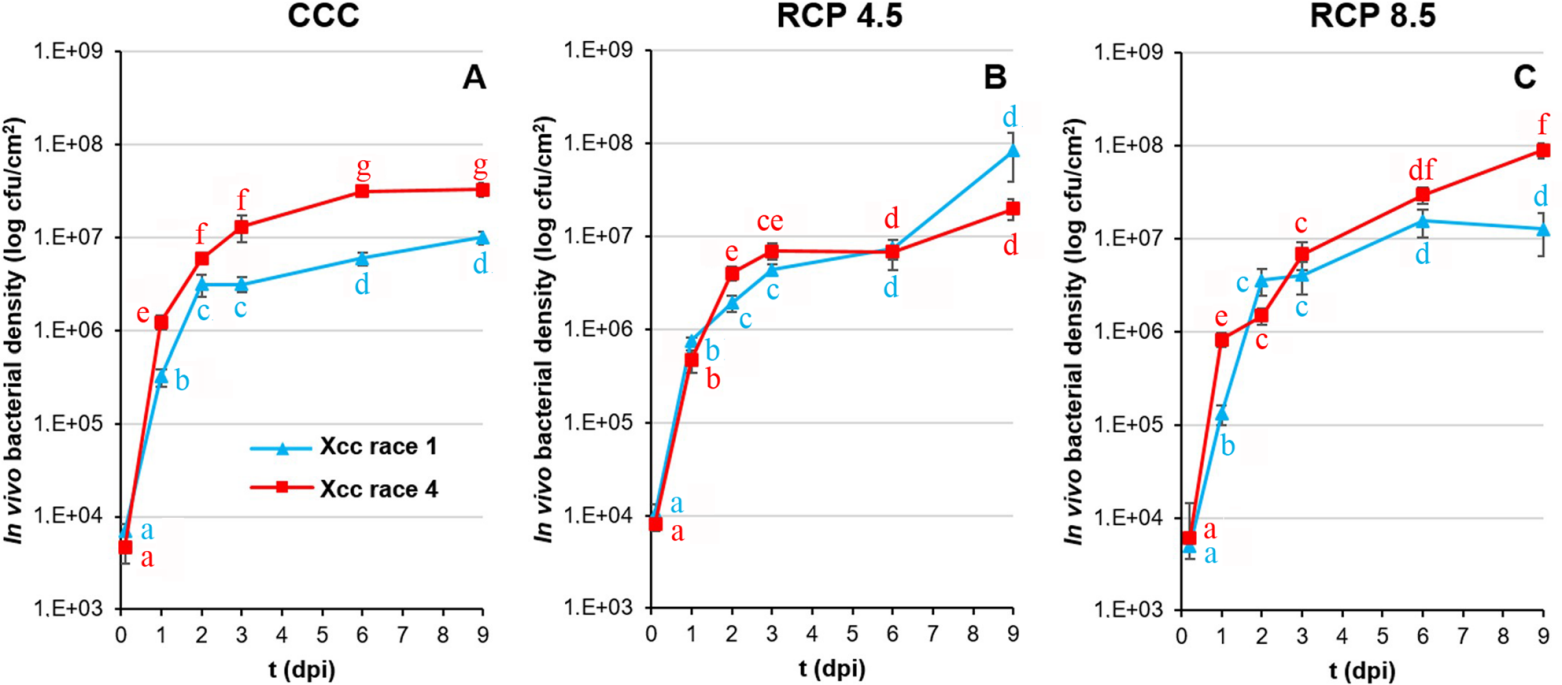
In vivo growth kinetics of Xcc in broccoli leaves under climate change conditions. Bacterial density found on leaves of plants grown under CCC (**A**), RCP 4.5 (**B**) and RCP 8.5 (**C**), determined in the area surrounding the inoculation points of broccoli leaves, and measured as colony forming units (cfu) per square centimetre from two hours after inoculation until 9 days post-inoculation (dpi). Data represent means ± standard error of six leaf discs (five in the case of RCP 8.5). Different lowercase letters indicate statistical differences at *p* < 0.05, according to one-way ANOVA followed by Tukey’s HSD test. Within each curve (same colour), different letters denote significant differences in bacterial growth between dpi for the corresponding Xcc race. For each dpi, different letters between colours indicate significant differences between Xcc races

In vivo growth of Xcc on plants cultivated under the three climatic conditions was evaluated in leaf areas arranged concentrically around the inoculation sites from 0 to 9 dpi (Fig. 2). Across 0–2 dpi, Xcc exponential growth was broadly similar among climate conditions; however, transient climate effects at individual early dpi were detected (notably 1 dpi for Xcc race 1, and 2 dpi for Xcc race 4; Suppl. Table 1). The bacterial concentration per leaf area was significantly higher for Xcc race 4 under CCC and RCP 8.5. In contrast, under RCP 4.5, both races reached the same bacterial density throughout the experiments. In consequence, only under CCC and RCP 8.5, race 4 reached a higher bacterial density than race 1.

### The regulation of stomatal movements in response to infection under climate change conditions depends upon the Xcc race

**Fig. 3:**
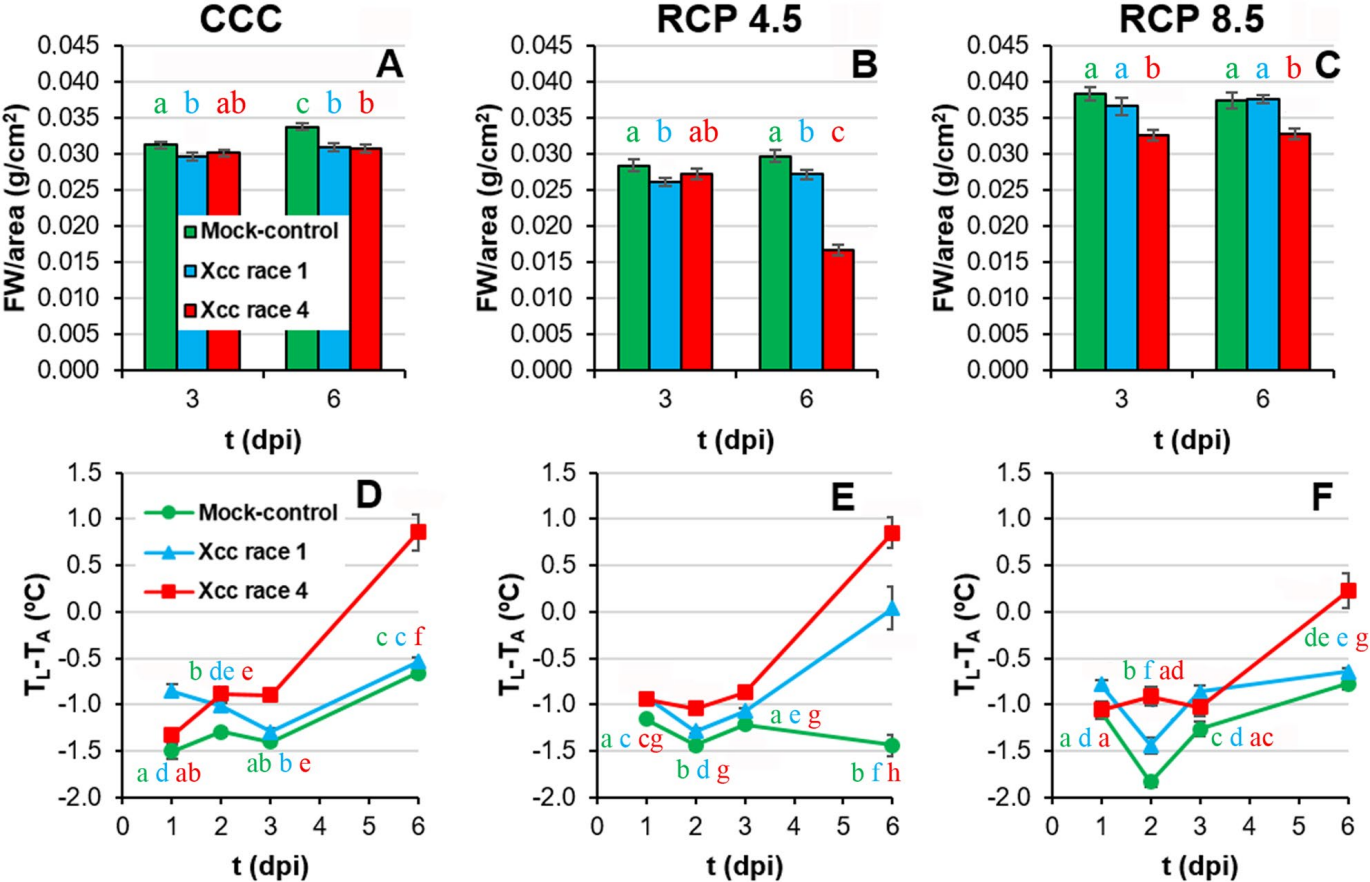
Leaf thickness and leaf temperature of Xcc-infected broccoli leaves of plants grown under climate change conditions. **A** to **C**: leaf thickness as measured by fresh weight (FW) per square centimetre; *n* = 48 for CCC and RCP 4.5, and *n* = 40 for RCP 8.5. **D** to **F**: leaf temperature corrected by ambient temperature (T_L_ – T_A_) of inoculated areas of broccoli leaves; *n* = 48 for CCC and RCP 4.5, and *n* = 16 for RCP 8.5. Far left panels (**A**, **D**) represent plants grown under CCC; middle panels (**B**, **E**) correspond to plants cultivated under RCP 4.5; whereas far right panels (**C**, **F**) represent plants grown under RCP 8.5. Graphs show means ± standard error. Statistical analysis was performed as described for Fig. 2, and significance letters should be interpreted accordingly

The water content of leaf tissues surrounding the inoculation sites (measured as FW per leaf area, Fig. 3A-C) decreased along the pathogenesis to a different extent, depending on the Xcc race and growth conditions. Under both CCC and RCP 4.5 environments, it was found a small but significant decrease in the water content of Xcc race 1 inoculated leaves compared to the controls as early as 3 dpi. At 6 dpi, Xcc race 1 and Xcc race 4 inoculated leaves lose similar water content relative to control plants at CCC. In contrast, under RCP 4.5 conditions, although both Xcc races caused a decrease in leaf water content at 6 dpi, areas inoculated with Xcc race 4 experienced the strongest decrease. On the other hand, under RCP 8.5, only Xcc race 4 caused a loss in water content at both 3 and 6 dpi. When comparing environmental scenarios for the same biological treatment and dpi, leaf water content differed among climates, with RCP 4.5 generally showing slightly lower FW per leaf area than CCC, whereas RCP 8.5 consistently exhibited the highest values (Suppl. Table 1). It is worth highlighting that plants were always well-watered to avoid any water restriction in the roots due to high transpiration rates under elevated temperature.

Leaf transpiration can be estimated using leaf temperature relative to air temperature (T_L_ – T_A_, Fig. 3D-F; Suppl. Table 1). T_L_ – T_A_ correlates positively with the transpiration rate and therefore the stomatal conductance. Across the three climate scenarios, T_L_–T_A_ values in mock-controls remained negative, but the magnitude and timing of T_L_–T_A_ increases in Xcc-infected leaves differed among climates. Thus, in the case of CCC (Fig. 3D), significant differences were found between control and Xcc race 1-infected leaves, with the latter being warmer at 1 and 2 dpi only. However, Xcc race 4-infected leaves showed progressively higher T_L_ – T_A_ values from 2 dpi onward. In the case of plants grown under RCP 4.5 conditions (Fig. 3E), Xcc-infected plants registered higher T_L_ – T_A_ values than control plants throughout the experiment, with the Xcc race 4-infected leaves reaching the highest temperatures. Finally, under RCP 8.5 conditions, Xcc race 1-infected leaves registered higher T_L_ – T_A_ values than controls from 1 to 3 dpi (Fig. 3F). Xcc race 4-infected leaves registered higher temperatures than control plants at 2 and 6 dpi. It is worth noticing that Xcc race 4–inoculated areas showed positive values for T_L_ – T_A_ at 6 dpi, regardless of the ambient conditions; however, the difference in terms of T_L_ – T_A_ between controls and Xcc race 4-infected leaves at RCP 8.5 is the smallest recorded at 6 dpi for any climatic condition tested. In contrast, Xcc race 1–inoculated areas showed values around 0 for this parameter at 6 dpi under RCP 4.5. Positive T_L_ – T_A_ values were attributed to the appearance of necrosis in the tissue.

### Xcc races impact differently on the redox state of leaves under climate change conditions

**Fig. 4:**
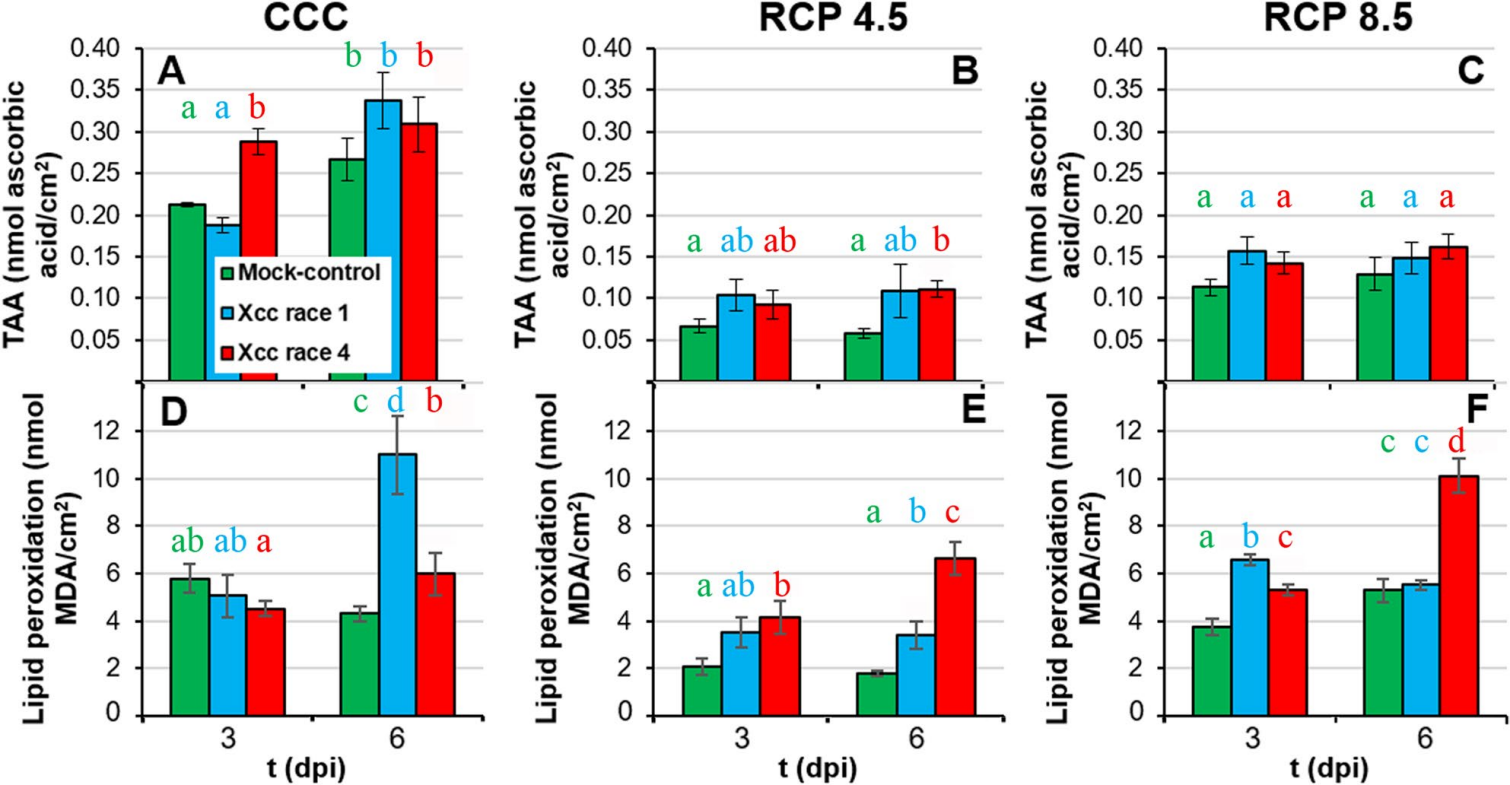
Redox state of Xcc-infected broccoli leaves of plants grown under climate change conditions. **A** to **C**: total antioxidant activity (TAA); **D** to **F**: lipid peroxidation of membranes. All measurements are referred to leaf area. Far left panels (**A**, **D**) represent plants grown under CCC; middle panels (**B**, **E**) correspond to plants cultivated under RCP 4.5; whereas far right panels (**C**, **F**) represent plants grown under RCP 8.5. Graphs show means ± standard error of *n* = 6 for CCC and RCP 4.5, and *n* = 5 for RCP 8.5. Statistical analysis was performed as described for Fig. 2, and significance letters should be interpreted accordingly

Oxidative stress was determined based on TAA and the extent of lipid peroxidation in cell membranes (Fig. 4; Suppl. Table 1). On one hand, regardless of the biological treatment, TAA was lower under RCP 4.5 and RCP 8.5 conditions relative to CCC (Fig. 4A-C). No significant differences were found between the TAA measured for Xcc race 1-infected leaves relative to controls at any dpi or environmental condition tested. Under CCC and RCP 4.5, TAA was higher in Xcc race 4-infected leaves relative to mock-controls at 3 and 6 dpi, respectively. However, no significant differences were found between infected and control plants under RCP 8.5 conditions. On the other hand, lipid peroxidation values differed among climate treatments. Plants grown at RCP 4.5 showed the lowest levels for mock-controls and Xcc race 1-infected leaves. Under CCC, Xcc infected leaves, particularly with race 1, showed higher lipid peroxidation than the corresponding control plants at 6 dpi (Fig. 4D). Under RCP 4.5 (Fig. 4E), lipid peroxidation was higher in inoculated leaves than in control plants: in Xcc race 1-inoculated leaves only at 6 dpi, and in Xcc race 4-inoculated leaves from 3 dpi. Finally, under RCP 8.5 (Fig. 4F), lipid peroxidation was higher upon infection at 3 dpi. In contrast, at 6 dpi only Xcc race 4 inoculated leaves exhibited a significantly higher lipid peroxidation than the corresponding control plants.

### The photosynthetic activity of plants is affected more severely by Xcc race 4

**Fig. 5:**
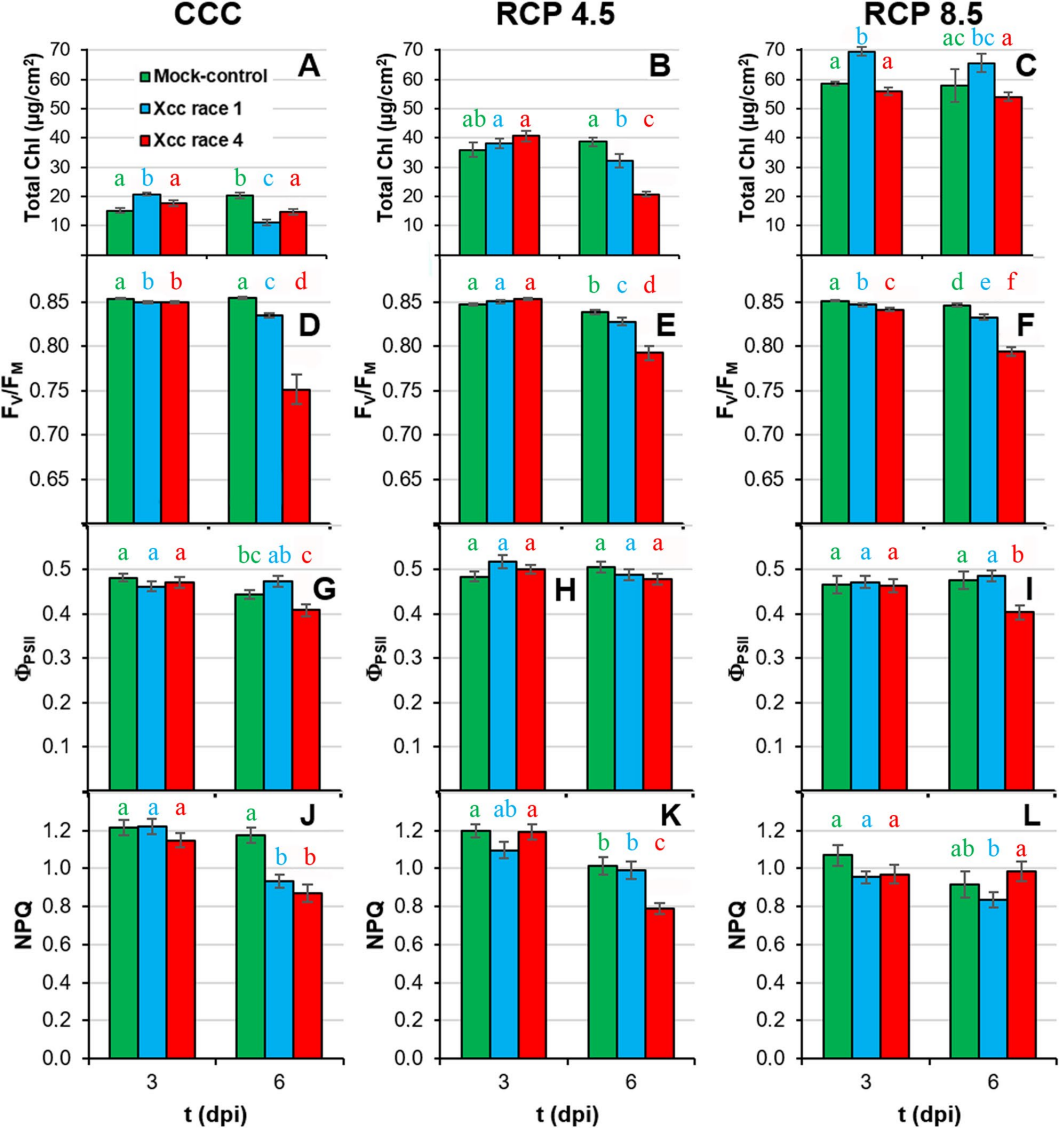
Primary metabolism of Xcc-infected broccoli leaves of plants grown under climate change conditions. **A** to **C**: total chlorophyll (Chl) content; **D** to **F**: maximum quantum yield of PSII (F_V_/F_M_): **G** to **I**: effective quantum yield of PSII (Φ_PSII_); **J** to **L**: non-photochemical quenching of PSII (NPQ); Far left panels (**A**, **D**, **G**, **J**) represent plants grown under CCC; middle panels (**B**, **E**, **H**, **K**) correspond to plants cultivated under RCP 4.5; whereas far right panels (**C**, **F**, **I**, **L**) represent plants grown under RCP 8.5. Graphs show means ± standard error of *n* = 32 samples, excepting for Chl content, where *n* = 6 for CCC and RCP 4.5, and *n* = 5 for RCP 8.5. Statistical analysis was performed as described for Fig. 2, and significance letters should be interpreted accordingly. Dpi: days post-inoculation

The oxidative stress caused by the phytopathogenesis has an impact on the integrity and functionality of the thylakoid membranes (Fig. 5; Suppl. Table 1), affecting the pigment composition of leaves. Increases in temperature and CO_2_ levels resulted in progressive increments in the amount of total Chl. At CCC, the total Chl content of Xcc race 1-infected leaves was higher than that measured in control leaves at 3 dpi (Fig. 5A). However, at 6 dpi, both Xcc infected leaves showed a lower Chl content relative to control plants. Similarly, under RCP 4.5 (Fig. 5B), Xcc infected leaves displayed lower Chl values than the control plants at 6 dpi. Finally, under RCP 8.5 conditions, the only significant difference between infected and control leaves was found at 3 dpi, when Xcc race 1-inoculated leaves showed higher values than mock-control leaves (Fig. 5C). These decrease in Chl content in inoculated leaves correlated with a permanent photodamage to PSII, as indicated by the decrease in its maximum efficiency (F_V_/F_M_). Even though no biologically significant differences were observed for F_V_/F_M_ at 3 dpi under any of the tested environmental conditions (Fig. 5D-F), as infection progressed, this parameter decreased in inoculated plants compared to control plants. Furthermore, at 6 dpi, Xcc race 4–inoculated leaves showed the most severe decrease of F_V_/F_M_, particularly under CCC. However, the quantum yield of PSII (Φ_PSII_, Fig. 5G-I) was not affected, indicating that this damage to PSII had little impact on PSII performance. Indeed, only the 6 dpi Xcc race 4-infected leaves of plants under the RCP 8.5 environment showed a significant decreased Φ_PSII_ value relative to control and Xcc race 1–inoculated plants (Fig. 5I). Furthermore, energy dissipated by non-photochemical processes (NPQ) decreased at 6 dpi in both Xcc race 1- and Xcc race 4-infected plants relative to control plants under CCC (Fig. 5J), and only in Xcc race 4 inoculated leaves in the case of RCP 4.5 (Fig. 5K); moreover, no significant differences were found between biological treatments under RCP 8.5 conditions (Fig. 5L).

### The secondary metabolism is activated in response to Xcc infection under climate change conditions

**Fig. 6:**
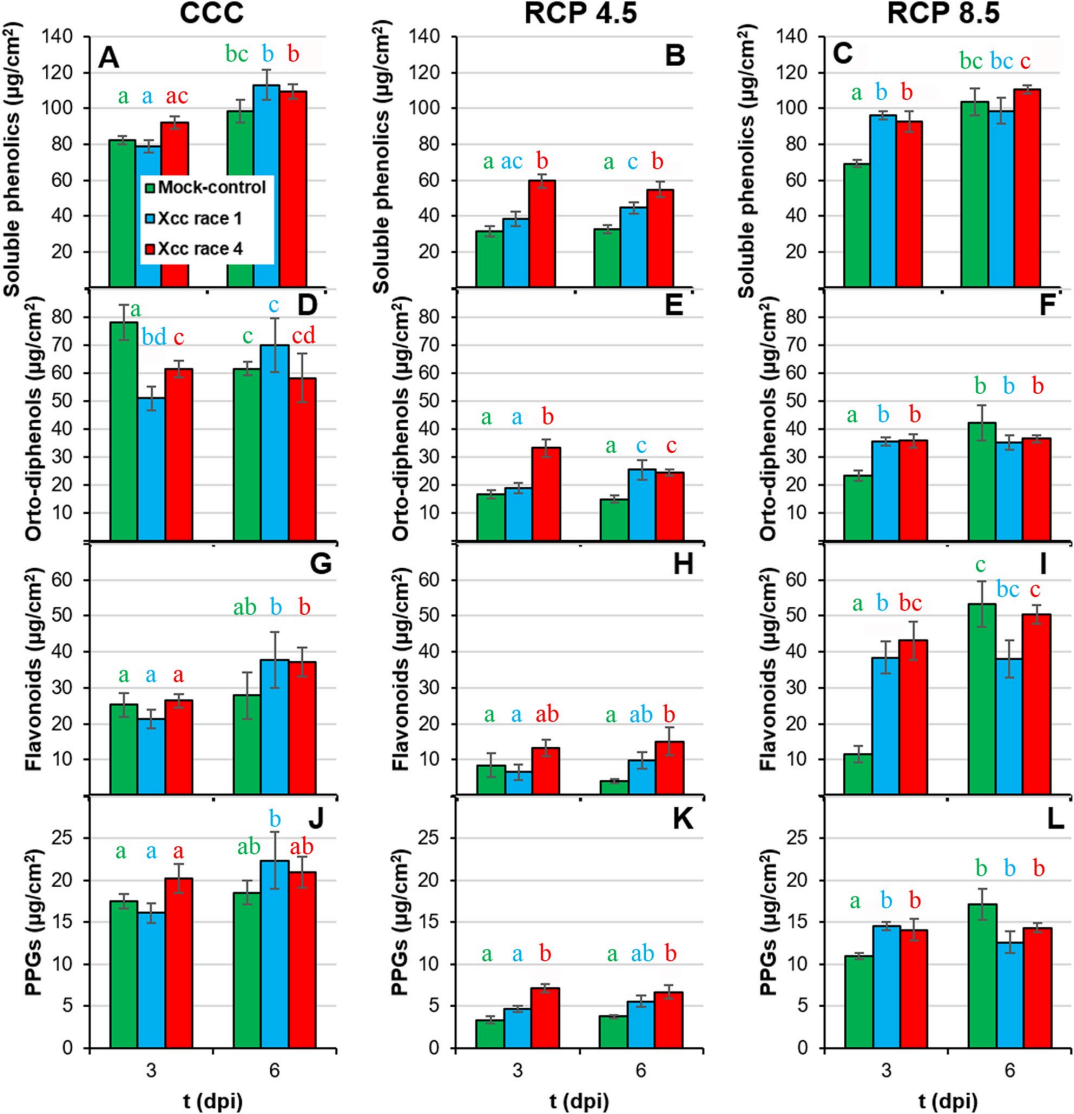
Secondary metabolism of Xcc-infected broccoli leaves of plants grown under climate change conditions I. **A** to **C**: leaf content on total soluble phenolic compounds; **D** to **F**: leaf content on ortho-diphenols; and **G** to **I**: leaf content on flavonoids; **J** to **L**: phenylpropanoid glycosides (PPGs). Far left panels (**A**, **D**, **G**, **J**) represent plants grown under CCC; middle panels (**B**, **E**, **H**, **K**) correspond to plants cultivated under RCP 4.5; while far right panels (**C**, **F**, **I**, **L**) represent plants grown under RCP 8.5. Graphs show means ± standard error of *n* = 6 for CCC and RCP 4.5, and *n* = 5 for RCP 8.5. Statistical analysis was performed as described for Fig. 2, and significance letters should be interpreted accordingly. Dpi: days post-inoculation.

Total soluble phenolics (emitting F440 and F520) were measured as products of secondary metabolism with important antioxidant properties, among other functions related to plant defence. In general terms, the greater magnitude change in the content of the measured phenolics was imposed by RCP 4.5 projection (Fig. 6; Suppl. Table 1). Under CCC (Fig. 6A), no statistically significant differences in total soluble phenolics were found between infected and control plants at any timepoint analysed. In contrast, under RCP 4.5 conditions, Xcc race 4-infected plants showed higher total soluble phenolics content than the corresponding control plants at both 3 and 6 dpi (Fig. 6B); while Xcc race 1–inoculated plants accumulated more soluble phenolics than control plants only at 6 dpi. Under RCP 8.5 conditions, plants infected with Xcc races 1 and 4 showed higher amounts of total soluble phenolic compounds than control plants only at 3 dpi (Fig. 6C). This increase in soluble phenolics in response to infection under climate change conditions could be due, at least in part, to an increase in the contents of ortho-diphenols, flavonoids and PPGs (Fig. 6D and L). Interestingly, ortho-diphenols showed a higher accumulation in plants under CCC conditions, compared to the RCP conditions. Whereas the infection caused a transient decrease at 3 dpi under CCC (Fig. 6D), under RCP conditions ortho-diphenols accumulated to higher levels than in control plants (Fig. 6E and F).

**Fig. 7:**
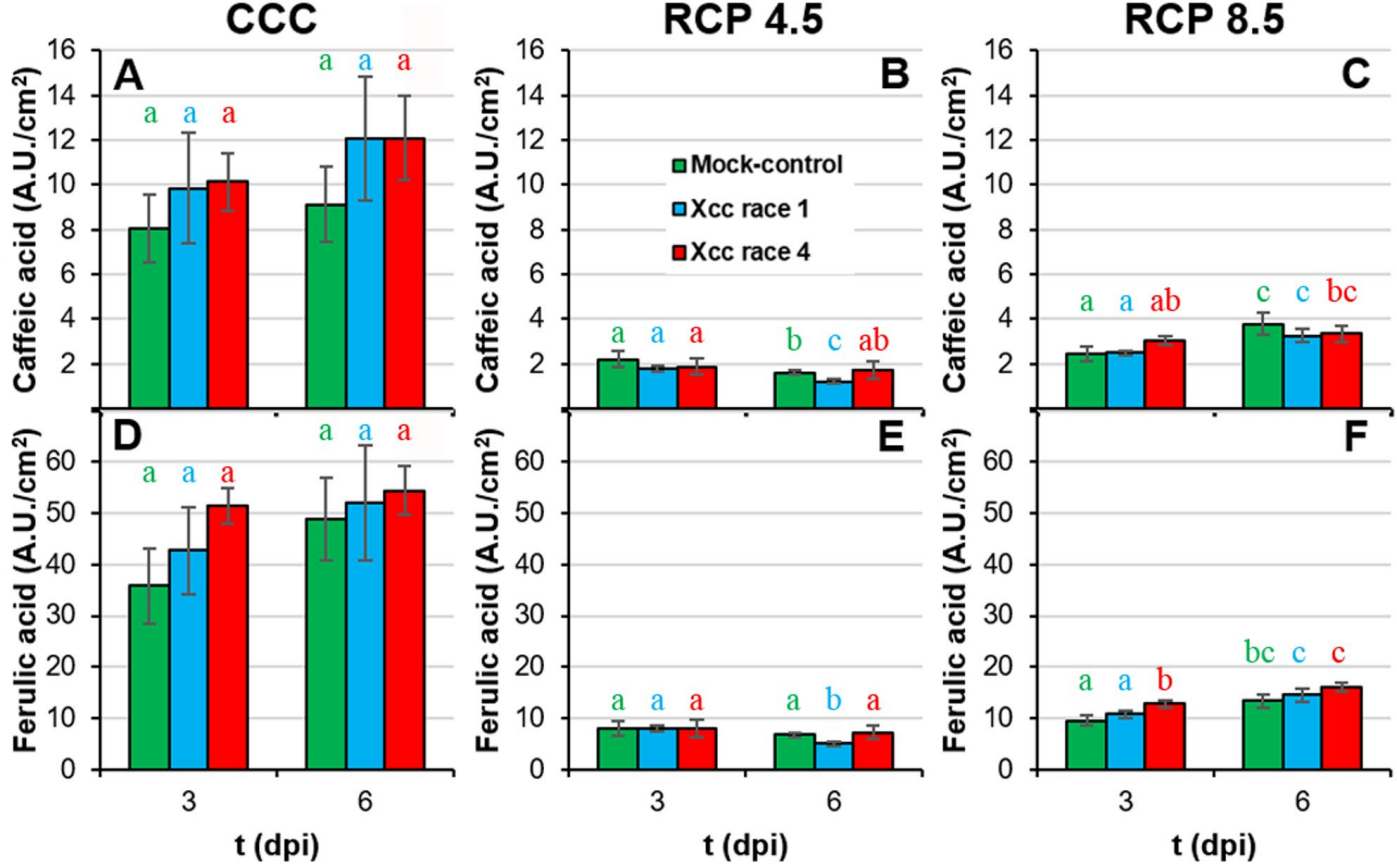
Secondary metabolism of Xcc-infected broccoli leaves of plants grown under climate change conditions II. Concentration per square centimetre of: **A** to **C**: caffeic acid; **D** to **F**: ferulic acid. Far left panels (**A**, **D**) represent plants grown under CCC; middle panels (**B**, **E**) correspond to plants cultivated under RCP 4.5; whereas far right panels (**C**, **F**) represent plants grown under RCP 8.5. Graphs show means ± standard error of *n* = 6 for CCC and RCP 4.5, and *n* = 5 for RCP 8.5. Statistical analysis was performed as described for Fig. 2, and significance letters should be interpreted accordingly

Among the phenylpropanoids, hydroxycinnamic acids such as caffeic and ferulic acid are important both as precursors of monolignols, monomers of lignin, and as antioxidants. Plants grown under RCP conditions displayed a severe decrease in the caffeic and ferulic acid content compared to CCC plants (Fig. 7A-F; Suppl. Table 1). However, the differences observed between infected and control plants were very small, regardless of the environmental conditions.

**Fig. 8:**
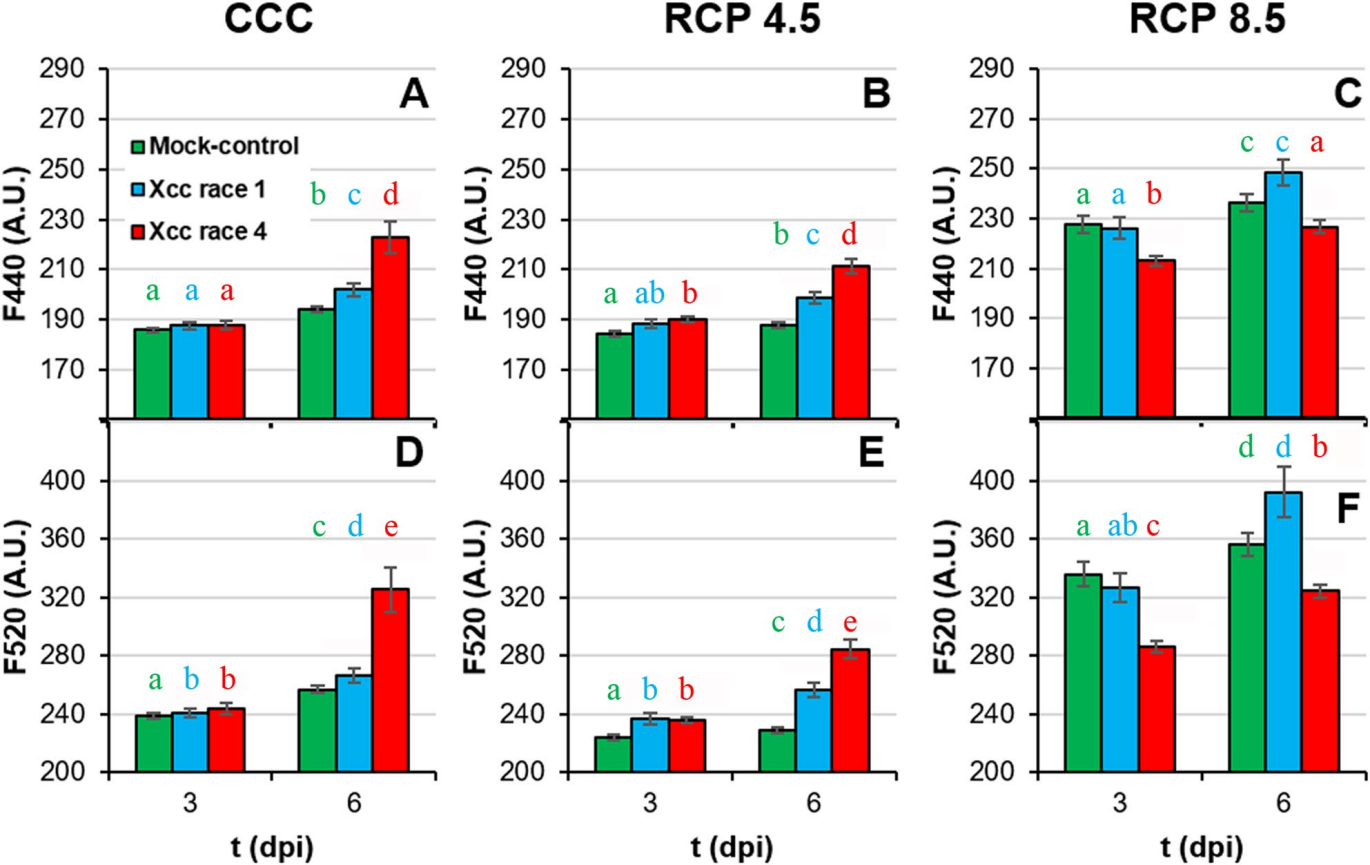
Secondary metabolism of Xcc-infected broccoli leaves of plants grown under climate change conditions III. Blue (F440; **A** to **C**) and green (F520; **D** to **F**) fluorescence emitted by inoculated areas of broccoli leaves. Far left panels (**A**, **D**) represent plants grown under CCC; middle panels (**B**, **E**) correspond to plants cultivated under RCP 4.5; whereas far right panels (**C**, **F**) represent plants grown under RCP 8.5. Graphs show means ± standard error of *n* = 48 for CCC and RCP 4.5, and *n* = 16 for RCP 8.5. Statistical analysis was performed as described for Fig. 2, and significance letters should be interpreted accordingly. Dpi: days post-inoculation

Phenolic compounds can be detected in vivo by autofluorescence (F440 and F520, Fig. 8; Suppl. Table 1). The RCP 8.5 projection enhanced the leaf autofluorescence over the other climatic conditions tested. Under CCC and RCP 4.5, an increase in F440 and F520 was observed for Xcc-infected plants relative to the corresponding control plants, particularly for Xcc race 4–inoculated plants at 6 dpi. In contrast, plant grown under RCP 8.5, displayed higher values of F440 and F520 than those grown under CCC or RCP 4.5. Furthermore, under RCP 8.5 only Xcc race 4-infected plants showed significantly lower F440 and F520 values at 3 and 6 dpi relative to the corresponding control plants.

**Fig. 9:**
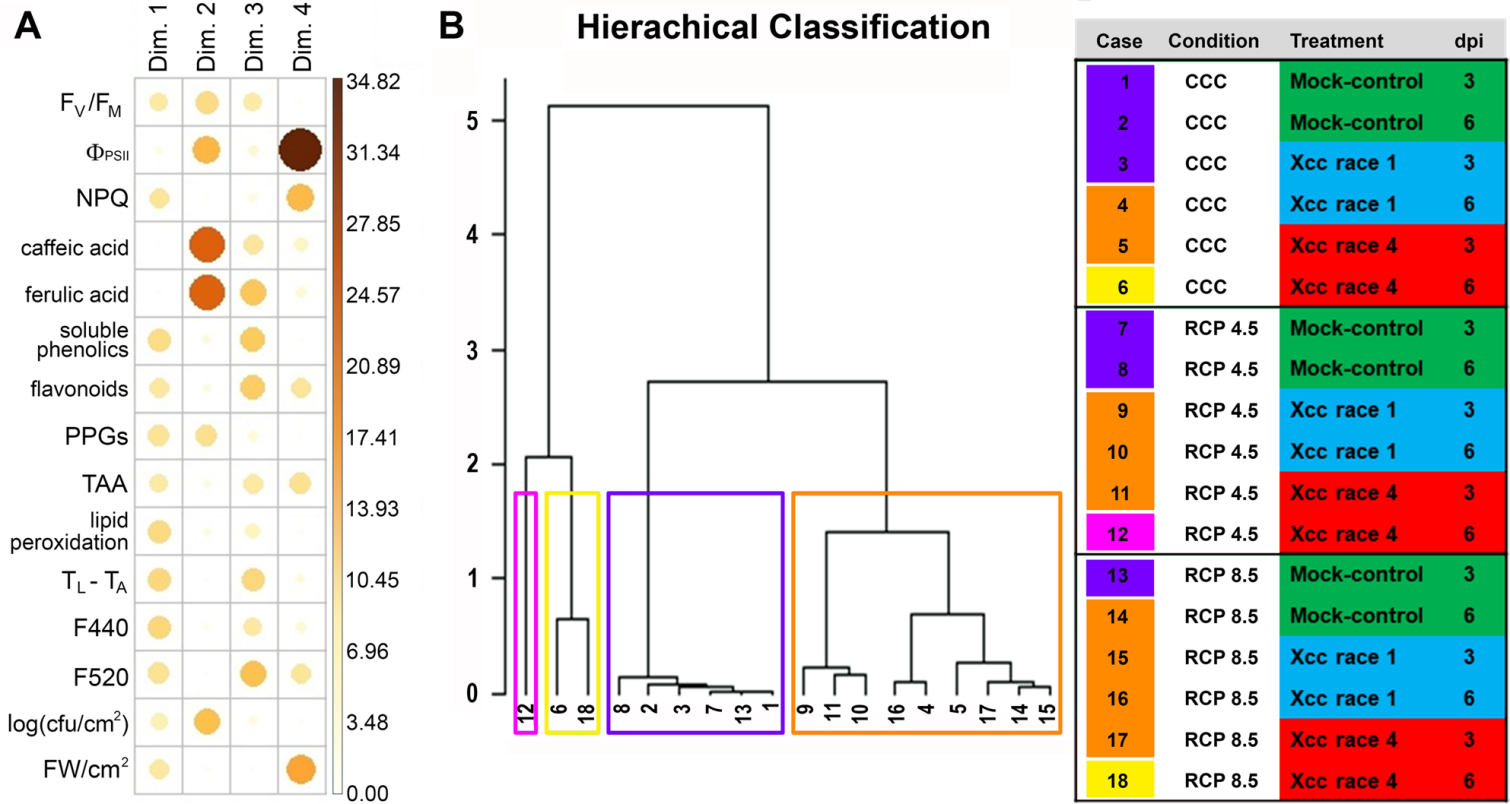
Hierarchical clustering of principal component (HCPC) analysis. **A**: Contributions of each of the variables used in the analysis to each of the axes of the principal component analysis (PCA), according to the colour scale on the right. **B**: Hierarchical classification of every treatment upon PCA, showing the obtained four groups, framed in different colours. The number indicates the treatment, listed in the table on the right. F_V_/F_M_: maximum quantum yield of PSII; Φ_PSII_: effective quantum yield of PSII; NPQ: non-photochemical quenching of PSII; PPGs: phenylpropanoids glycosides; TAA: total antioxidant activity; T_L_ – T_A_: leaf temperature corrected by ambient temperature; F440: blue fluorescence; F520: green fluorescence; cfu: colony forming units; FW: fresh weight

To further analyse the overall effect of infection by the two Xcc races and under the three environmental conditions studied, a principal component analysis (PCA) followed by a hierarchical clustering (Fig. 9, Suppl. Figure 1) was performed on physiological parameters. These parameters were selected prior to the PCA to avoid redundancy of the data. In the hierarchical clustering, control samples at 3 and 6 dpi under the three environmental conditions clustered together, except for 6 dpi under RCP 8.5. Xcc race 1–inoculated samples at 3 dpi under CCC were also grouped with control samples. The second group included all Xcc race 1–inoculated plants at 3 and 6 dpi under every ambient condition (with the exception already mentioned), plus Xcc race 4–inoculated plants at 3 dpi. Finally, Xcc race 4–inoculated plants at 6 dpi appeared in two independent groups, indicating large differences in the effects of Xcc race 1 and race 4 on plant physiology. According to this clustering, the response of broccoli plants to Xcc race 1 and race 4 was similar at early stages of the infection, regardless of the environmental conditions. However, as the infection progressed, differences appeared between races. Under milder conditions (CCC and RCP4.5), Xcc race 4 proved to be more virulent than race 1. However, under more extreme conditions, Xcc race 1 caused the most severe symptoms in the long term.

## Discussion

Human activities have increased greenhouse gas emissions such as CO_2_, leading to the so-called climate change, which is believed to have unprecedented effects on all living organisms. The fitness of plants, as sessile individuals, is particularly affected by alterations in environmental variables, such as temperature and CO_2_ concentrations. Indeed, plants have evolved a wide range of adaptive responses, involving adjustments in their metabolism and development, in order optimise their capacity for energy acquisition and resource utilisation to cope with unfavourable conditions [36]. Furthermore, climate change also significantly modifies the dynamics of organisms in interaction with plants. Thus, plant pathogens will undergo alterations in their virulence and spread, which challenges plant defence mechanisms against them, as well as the effectiveness of our disease management strategies. For these reasons, some authors, like [37] have urged researchers to consider multifactorial approaches in their studies in order to reproduce more realistic scenarios and to improve the applicability of their findings.

**Fig. 10:**
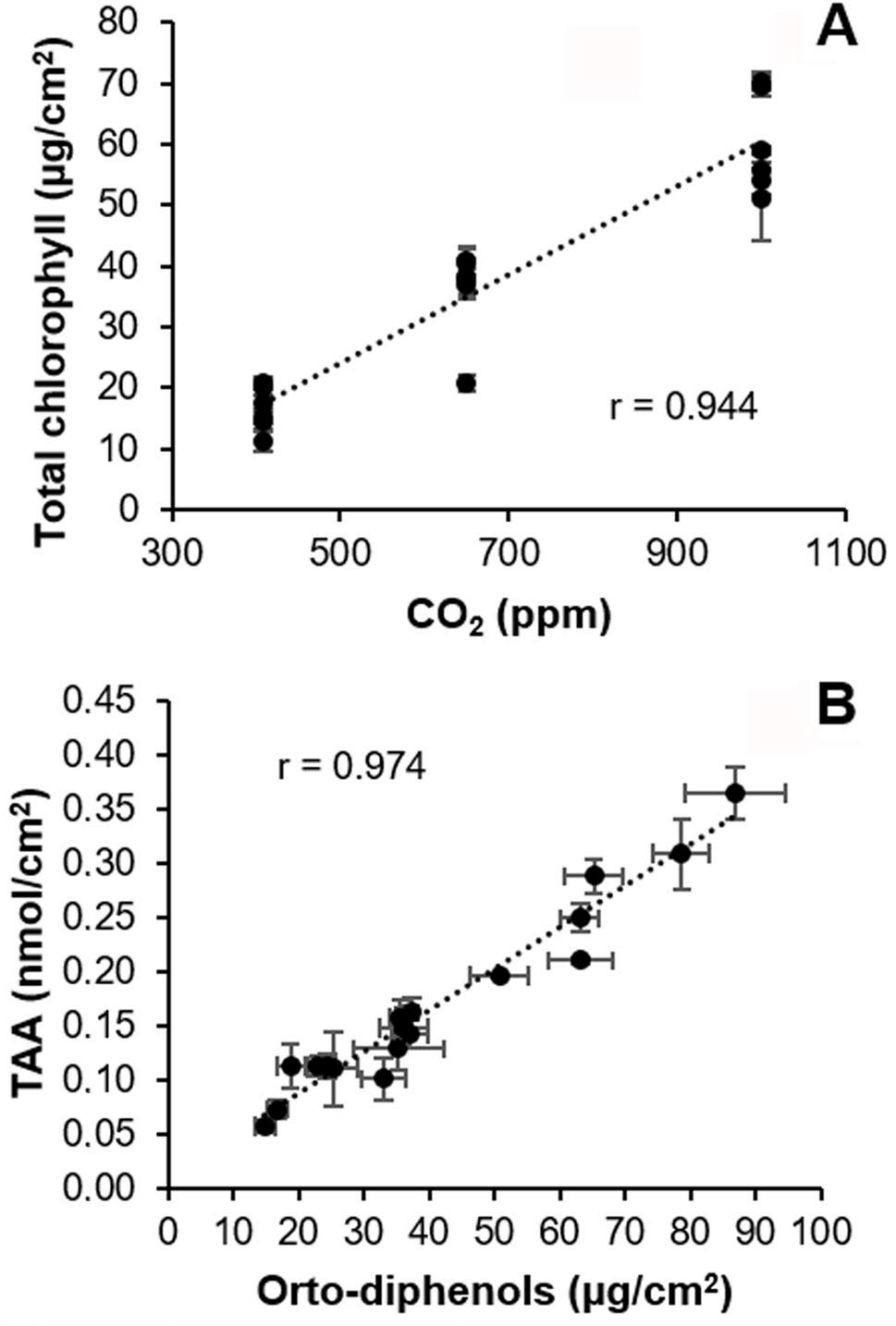
Correlation between several measured parameters. **A**: correlation between total chlorophyll content and CO_2_ concentration provided during plant growth; **B**: correlation between total antioxidant activity (TAA) and concentration of ortho-diphenols. *N* = 18. Graphs show means ± standard error. Linear correlation line is shown for each graph, as well as the Pearson correlation coefficient (r) at *p* < 0.01 is shown for every couple of variables

Moderate increases in ambient temperatures and/or CO_2_ levels enhance plant growth. Indeed, broccoli plants grown in RCP 4.5 environments activated adaptive mechanisms that maintained plant fitness at levels similar to those grown under CCC ([8]; this work). However, larger increases in ambient temperatures and/or CO_2_ may hinder plant growth [2, 38]. Actually, responses to CO_2_ ambient concentration is complex, since Chl content, as an indicator of photosynthetic activity, positively correlates with ambient CO_2_ (Fig. 10A), but other physiological traits indicate that extreme weather conditions overcome adaptation capability of broccoli plants. Such was the case of broccoli plants grown at RCP 8.5 condition, under which plant development and physiology underwent severe alterations that seem to be insufficient to maintain plant fitness. These results are in agreement with those previously reported for *brassicas* such as broccoli [8, 39] and oilseed rape plants (*Brassica napus*) [40, 41]. The inhibition of plant growth under supra-optimal temperature and CO_2_ values is the result of photosynthetic deficiencies (Fig. 5; Suppl. Table 1) [2, 38, 42]. Results here presented are in agreement with those published by [8], where the downregulation of primary metabolism of broccoli plants grown under RCP 4.5 and, more pronouncely under RCP 8.5 conditions, were described. High temperatures may directly affect PSII efficiency and growth of plants through different processess such as Chl content decreases or changes in membranes properties [43, 44]. However, no Chl content diminution or lipid peroxidation of membranes were observed in broccoli plants due to climate change ([8]; Figs. 4A-C and 5A-C), suggesting that these factors are not directly responsible for this photosynthetic decrease. The explanation could be related to the observed decrease in stomatal gas exchange, suggested by the increase in leaf temperature (Fig. 3D-F; Suppl. Table 1). It is known that, in C_3_ plants, high intercellular CO_2_ concentrations decrease stomatal aperture, thus incrementing leaf temperature and decreasing the net photosynthesis rate [44]. Moreover, although global warming is increasing VPD (Table 1), plants often counteract this rise in evaporative demand by actively restricting stomatal aperture, ultimately reducing water loss [45] (Fig. 3A-C; Suppl. Table 1). Furthermore, the decrease in plant growth under climate change conditions points to a prioritisation of defence mechanisms over plant growth [8]. Although decreases in secondary metabolism compounds have been measured (Figs. 6 and 7; Suppl. Table 1), these could be used to: (i) produce lignins, macromolecules with a marked defensive character which fluorescence in the blue and green regions of the spectrum (Fig. 8; Suppl. Table 1; [46, 47]); (ii) promote an increase in the non-enzymatic antioxidant pool and thereby a decrease in the lipid peroxidation. Particularly ortho-diphenols could be contributing to increased antioxidant capacities, as suggested by their high correlation (Fig. 10B). Additionally, rising temperatures and CO_2_ levels may also compromise the glucosinolate-myrosinase system, a central chemical defence strategy of the Brassicaceae family against stress [48]. However, glucosinolate concentrations were not measured as the biochemical analyses were specifically designed on the metabolites contributing to the fluorescence signals described here.

Climate change affects plant pathogens physiology and dynamics [49]. As an example, exposure to high temperatures causes a significant reduction in the viability of Xcc [50]. Indeed, RCP 8.5 conditions prevent Xcc growth in vitro [20]. Thus, the plant appears to exert a significant influence on Xcc proliferation. Indeed, the clustering in Fig. 9 suggested that the ambient conditions impact differently depending on the race, and so the timing and severity of the pathology.

Aside from climate change, Xcc has evolved sophisticated strategies to manipulate plant physiology, compromising crucial processes that are essential for host health and development [51]. Under CCC, Xcc race 4 is the most virulent strain, particularly noticeable at late phases of the infection process. (Fig. 1). In line with results here presented, it has been reported that infection by Xcc reduces both biomass and photosynthesis (Fig. 5D, G) in the aerial parts of broccoli seedlings, modifying their water content in a temporally dependent fashion (Fig. 3A) [52]. Xcc infection also caused a stomatal closure (Fig. 3D) and a Chl degradation (Fig. 5A), that could be related to the reduced activity of the PSII reaction centres, as shown by decreases in F_V_/F_M_, Φ_PSII_ and NPQ (Fig. 5D, G, J). In the case of cabbage (*B. oleracea* var. *capitata*), the registered decrease in photosynthesis during Xcc infection is not related to stomatal factors [53]. Furthermore, damage to membranes caused by lipid peroxidation induced by Xcc infection in broccoli plants (Fig. 4D) could also be responsible for the metabolic alterations observed. However, maintaining reactive oxygen species (ROS) homeostasis may represent a crucial mechanism for broccoli plants to mitigate Xcc infection. Actually, during the broccoli response to Xcc infection, most of the proteins whose accumulation levels decreased are involved in energetic metabolism, pointing to a reallocation of resources toward defence [54]. Thus, in broccoli-Xcc infected plants, non-enzymatic pathways appeared to be important for maintaining TAA levels similar to control plants (Fig. 4A). Indeed, at the late phases of Xcc infection, broccoli plants tended to accumulate soluble phenolics, with high antioxidant activity (Fig. 6A), and to keep the levels of orto-diphenols, flavonoids, PPGs (Fig. 6D, G, J), caffeic and ferulic acids (Fig. 7A, D) similar to control plants. The non-enzymatic pathways to scavenge ROS are also important in cabbage [55]. In these plants, Xcc promoted phenolic acid biosynthesis (including ferulic and caffeic acids), thus mitigating ROS accumulation and alleviating bacterial-induced damage [56]. Cabbage plants infected with Xcc showed similar flavonoids content than the control plants [57]. Moreover, phenolic compounds such as caffeic and ferulic acid are precursors of lignins. As already discussed, lignins emit fluorescence at 440 and 520 nm. Thus, increases in F440, and mainly F520, were registered in Xcc-infected broccoli plants (Fig. 8A, D), as it was also the case of Xcc-infected cabbage plants [58].

Since all plants were inoculated at the same phenological stage (BBCH 14) under all climatic condition, it is unlikely that developmental differences in host susceptibility (ontogenic resistance) account for the physiological differences observed among the biological treatments. Conversely, the complex interplay between the host plant, the bacterial pathogen and the environmental conditions seems to determine the progression and severity of the infection. Thus, climatic variables would also aggravate the intensity of diseases caused by *Xanthomonas* spp [11, 49, 59]. The extent of such intensification depends on the magnitude of the variation in temperature and CO_2_ concentration [60]. For example, barley is more vulnerable to *Blumeria graminis* f. sp. *hordei* when the plants are grown under elevated temperature and CO_2_ [61]. Arabidopsis plants grown at 30 °C are more susceptible to *Pseudomonas syringae* pv. *tomato* DC3000 than those plants grown at 23 °C [62]. In pepper, resistance to *Xanthomonas* is weakened under high temperatures [63]. Moreover, climate change amplifies the detrimental impact of Xcc infection on oilseed rape plants, causing a premature onset of Xcc symptoms linked to oxidative stress and modifications in pigment composition [20]. This is in line with results obtained in this work. Under climate change conditions, the elevated levels of lipid peroxidation observed in Xcc-infected broccoli leaves at advanced stages (Fig. 4) provide strong evidence that ROS accumulation induced by the pathogen contributes significantly to tissue damage. Thus, plant growth and photosynthesis measured in terms of F_V_/F_M_ and Φ_PSII_ (Fig. 5) were severely affected by the combination of abiotic and biotic stresses. The most stressful combination was RCP 8.5 conditions and Xcc race 4 infection. Under RCP 8.5 conditions, the decrease in photosynthetic efficiency was not related to a decrease in Chl content (Fig. 5C) or to more pronounced stomatal closure than that registered in plants grown under CCC (Fig. 3F), suggesting that other factors are contributing to the decline in photosynthesis. Promoting secondary metabolism (Figs. 6, 7 and 8) over primary metabolism may be the answer to this question, as discussed above. As an example in cabbage, the combined stress of Xcc and drought resulted in an accumulation of flavonoids respecting to single stresses, while Chl content remained unchanged [58]. On the other hand, glucosinolates are hydrolysed by myrosinases following a pathogen attack, producing bioactive products with antimicrobial properties [48]. As mentioned above, future climate scenarios, particularly RCP 8.5, could cause an imbalance in glucosinolate-based defences favouring Xcc infection. Therefore, more specific metabolomics analyses are required in future studies.

Although both Xcc races successfully colonized broccoli leaves under all climate scenarios, differences in bacterial growth kinetics suggest race-specific responses to environmental cues. The degree of Xcc symptoms intensification due to climate change greatly depends on the magnitude of temperature and CO₂ concentration variation, as discussed above, rather than the pathogenic biomass accumulated in tissues [Fig. 2; [64], which is particularly true for pathogens invading vascular tissues [65]. Indeed, both climate change conditions exacerbated the infection caused by both strains of Xcc, with this being most evident under the RCP 8.5 condition (Fig. 1). However, the virulence of both strains differed according to the climatic condition analysed with no correlation with the number of cfus recovered from the affected tissues (Fig. 2). Under CCC and RCP 4.5, Xcc race 4 exhibited faster symptom development and earlier peak bacterial titters, suggesting enhanced virulence under milder conditions. Conversely, under RCP 8.5, race 1 demonstrated a surprising increase in aggressiveness and growth, surpassing race 4 at longer infection times. These shifts may reflect differential thermal optima or CO₂ responsiveness between races, a topic largely unexplored in current phytopathology literature. As such, the results here presented point to the critical role of host and pathogen plasticity in modelling disease outcomes under projected climate conditions.

## Conclusions

Future environmental conditions, RCP 4.5 and more pronouncedly RCP 8.5, would affect plant physiology and exacerbate the severity of black rot caused by Xcc races 1 and 4. Photosynthetic efficiency would decline under future climatic conditions, leading to metabolic disruptions, such as unbalanced production of pigments, antioxidants and secondary metabolites. Extreme climate change condition RCP 8.5 could markedly reduce plant resilience; however, broccoli plants showed an adaptive response to moderate abiotic stress. In spite of Xcc growth being challenged by environmental cues, the plant efforts to counteract the infection were not sufficient. Thus, combined climate and pathogen stress enhanced stomatal closure and oxidative damage, as well as promoted the synthesis of antioxidant phenolic compounds, indicating a defence-oriented metabolic reprogramming.

## Supplementary Information

Supplementary Material 1: Sup. Figure 1: Representation of the quality of the principal component analysis (PCA) shown in Fig. 9 (A) Squared cosine (variable cos2) value of each variable used indicating the quality of its representation by the PCA. (B) Biplot representing each sample (numbered dots) in a two-dimensional space according to the PCA and the contributions of each variable (vectors) to it. (C) Scree plot showing the explained variance (eigenvalue) of each principal component in descending order.

Supplementary Material 2.

## Data Availability

Data will be made available on request.
