# Supplementary figures and images for: Broccoli plants exposed to the combined threat of climate change and bacterial infection
Source: BMC Plant Biol. 2026 Apr 9;26:846. doi: 10.1186/s12870-026-08704-6 (PMC13173881; doi:10.1186/s12870-026-08704-6)

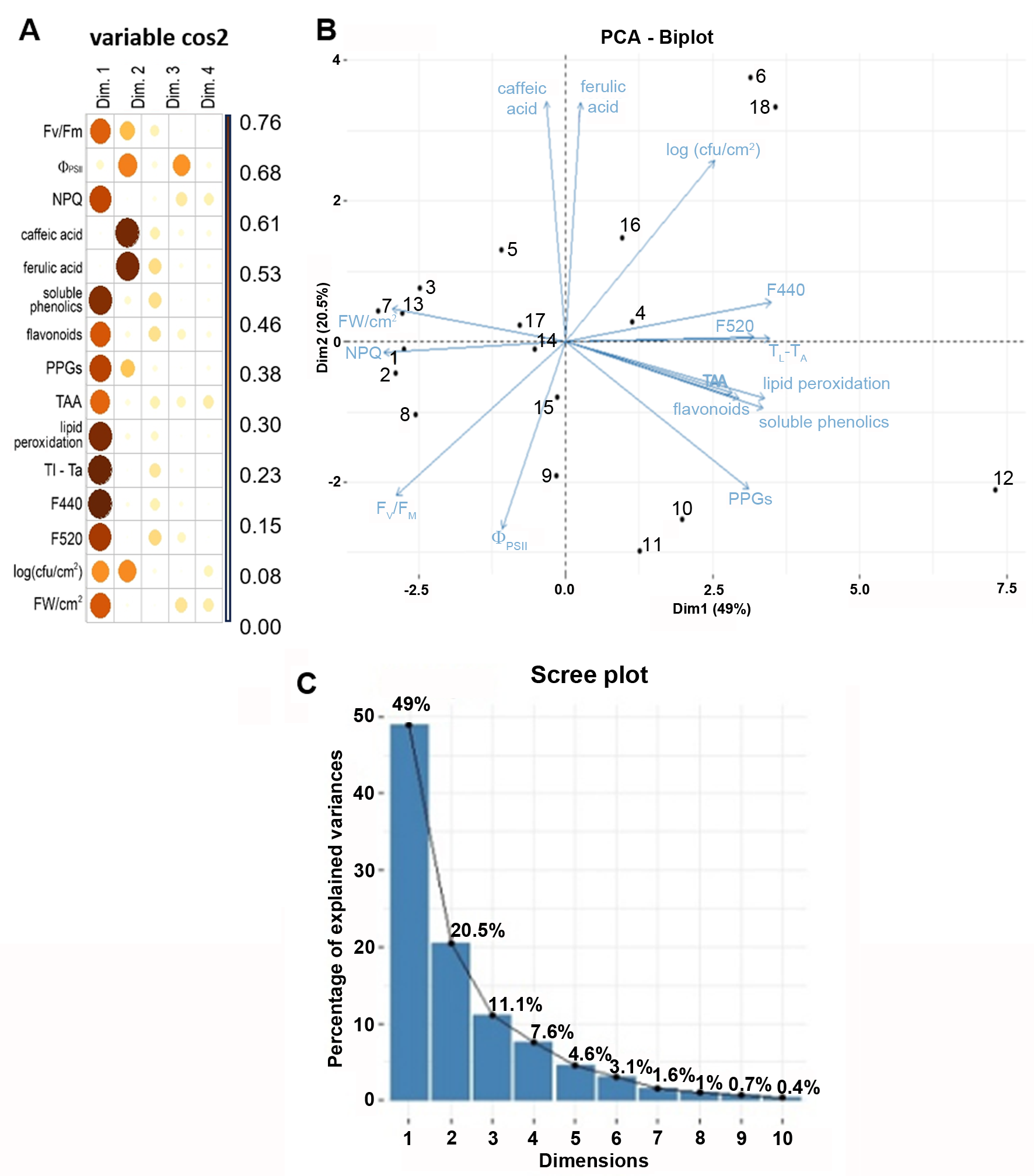

Supplement: Supplementary file 1 — Supplementary Material 1: Sup. Figure 1: Representation of the quality of the principal component analysis (PCA) shown in Fig. 9 (A) Squared cosine (variable cos2) value of each variable used indicating the quality of its representation by the PCA. (B) Biplot representing each sample (numbered dots) in a two-dimensional space according to the PCA and the contributions of each variable (vectors) to it. (C) Scree plot showing the explained variance (eigenvalue) of each principal component in descending order. [file 12870_2026_8704_MOESM1_ESM.tif]
