# Supplementary material for: Broccoli plants exposed to the combined threat of climate change and bacterial infection
Source: BMC Plant Biol. 2026 Apr 9;26:846. doi: 10.1186/s12870-026-08704-6 (PMC13173881; doi:10.1186/s12870-026-08704-6)
Supplement: Supplementary file 2 — Supplementary Material 2. [file 12870_2026_8704_MOESM2_ESM.docx]

**Supplementary Table 1 (I)** Effects of climate treatments (CCC, RCP 4.5 and RCP 8.5) on several parameters measured in mock-control and *Xanthomonas campestris* pv. *campestris* (Xcc) race 1- and race 4-infected plants. Different uppercase letters indicate statistical differences between climate treatments within each biological treatment and days post-inoculation (dpi) (i.e., they compare climates across figure panels) at *p* < 0.05, according to one-way ANOVA followed by Tukey’s HSD test. CCC: current climate conditions; cfu: colony forming units; F440, blues fluorescence; F520, green fluorescence; Φ_PSII_, effective quantum yield of photosystem II; F_V_/F_M_, maximum quantum yield of photosystem II; FW: fresh weight; MDA, malondialdehyde; NPQ, non-photochemical quenching; PPGs, phenylpropanoid glycosides; RCP 4.5 and RCP 8.5: Representative Concentration Pathways 4.5 and 8.5; dpi: days post-infection; TAA, total antioxidant activity; A.U., arbitrary units.

|  | Biological treatment | Mock-control | | | Xcc race 1 | | | Xcc race 4 | | |
| --- | --- | --- | --- | --- | --- | --- | --- | --- | --- | --- |
|  | Climate treatment | CCC | RCP 4.5 | RCP 8.5 | CCC | RCP 4.5 | RCP 8.5 | CCC | RCP 4.5 | RCP 8.5 |
| Bacterial density (cfu/cm^2^) | 0 dpi | / | / | / | A | A | A | A | A | A |
|  | 1 dpi | / | / | / | A | B | C | A | B | A |
|  | 2 dpi | / | / | / | A | A | A | A | A | B |
|  | 3 dpi | / | / | / | A | A | A | A | A | A |
|  | 6 dpi | / | / | / | A | A | A | A | B | A |
|  | 9 dpi | / | / | / | A | A | A | A | A | B |
| FW/área (g/cm^2^) | 3 dpi | A | B | C | A | B | C | A | B | C |
|  | 6 dpi | A | B | C | A | B | C | A | B | C |
| Temperature (ºC) | 1 dpi | A | B | B | A | A | A | A | B | C |
|  | 2 dpi | A | B | C | A | B | C | A | B | A |
|  | 3 dpi | A | B | B | A | B | C | A | A | B |
|  | 6 dpi | A | B | A | A | B | A | A | A | B |
| TAA (nmol ascorbic acid/cm^2^) | 3 dpi | A | B | C | A | B | C | A | B | C |
|  | 6 dpi | A | B | C | A | B | B | A | B | C |
| Lipid peroxidation (nmol MDA/cm^2^) | 3 dpi | A | B | C | A | A | B | A | A | B |
|  | 6 dpi | A | B | C | A | B | C | A | A | B |
| Total Chl content (µg/cm^2^) | 3 dpi | A | B | C | A | B | C | A | B | C |
|  | 6 dpi | A | B | C | A | B | C | A | B | C |
| F_V_/F_M_ | 3 dpi | A | B | A | A | A | A | A | B | C |
|  | 6 dpi | A | B | C | A | A | A | A | B | B |
| Φ_PSII_ | 3 dpi | A | A | A | A | B | A | AB | B | A |
|  | 6 dpi | A | B | AB | A | A | A | A | B | A |
| NPQ | 3 dpi | A | A | B | A | B | C | A | A | B |
|  | 6 dpi | A | B | B | AB | A | B | AB | A | B |

**Supplementary Table 1 (II)** **Supplementary Table 1 (I)** Effects of climate treatments (CCC, RCP 4.5 and RCP 8.5) on several parameters measured in mock-control and *Xanthomonas campestris* pv. *campestris* (Xcc) race 1- and race 4-infected plants. Different uppercase letters indicate statistical differences between climate treatments within each race and days post-inoculation (dpi) (i.e., they compare climates across figure panels) at *p* < 0.05, according to one-way ANOVA followed by Tukey’s HSD test. CCC: current climate conditions; cfu: colony forming units; F440, blues fluorescence; F520, green fluorescence; Φ_PSII_, effective quantum yield of photosystem II; F_V_/F_M_, maximum quantum yield of photosystem II; FW: fresh weight; MDA, malondialdehyde; NPQ, non-photochemical quenching; PPGs, phenylpropanoid glycosides; RCP 4.5 and RCP 8.5: Representative Concentration Pathways 4.5 and 8.5; dpi: days post-infection; TAA, total antioxidant activity; A.U., arbitrary units.

|  | Biological treatment | Mock-control | | | Xcc race 1 | | | Xcc race 4 | | |
| --- | --- | --- | --- | --- | --- | --- | --- | --- | --- | --- |
|  | Climate treatment | CCC | RCP 4.5 | RCP 8.5 | CCC | RCP 4.5 | RCP 8.5 | CCC | RCP 4.5 | RCP 8.5 |
| Soluble phenolics (µg/cm^2^) | 3 dpi | A | B | C | A | B | C | A | B | A |
|  | 6 dpi | A | B | A | A | B | A | A | B | A |
| Orto-diphenols (µg/cm^2^) | 3 dpi | A | B | C | A | B | C | A | B | B |
|  | 6 dpi | A | B | C | A | B | C | A | B | C |
| Flavonoids (µg/cm^2^) | 3 dpi | A | B | B | A | B | C | A | B | C |
|  | 6 dpi | A | B | C | A | B | A | A | B | C |
| PPGs (µg/cm^2^) | 3 dpi | A | B | C | A | B | A | A | B | C |
|  | 6 dpi | A | B | A | A | B | C | A | B | C |
| Caffeic acid (A.U./cm^2^) | 3 dpi | A | B | B | A | B | C | A | B | C |
|  | 6 dpi | A | B | C | A | B | C | A | B | C |
| Ferulic acid (A.U./cm^2^) | 3 dpi | A | B | B | A | B | C | A | B | C |
|  | 6 dpi | A | B | C | A | B | C | A | B | C |
| F440 (A.U.) | 3 dpi | A | A | B | A | A | B | A | A | B |
|  | 6 dpi | A | B | C | A | A | B | A | B | A |
| F520 (A.U.) | 3 dpi | A | B | C | A | A | B | A | B | A |
|  | 6 dpi | A | B | C | A | A | B | A | B | A |
